# Impact of marital status on survival in patients with stage 1A NSCLC

**DOI:** 10.18632/aging.203838

**Published:** 2022-01-19

**Authors:** Liu Huang, Shu Peng, Chenyu Sun, Lian Chen, Qian Chu, Sudip Thapa, Vanisha Chummun, Lu Zhang, Peng Zhang, Eric L. Chen, Ce Cheng, Yuan Chen

**Affiliations:** 1 Department of Oncology, Tongji Hospital, Tongji Medical College, Huazhong University of Science and Technology, Wuhan 430030, Hubei, P.R. China; 2 Department of Thoracic Surgery, Tongji Hospital, Tongji Medical College, Huazhong University of Science and Technology, Wuhan 430030, Hubei, P.R. China; 3 AMITA Health Saint Joseph Hospital Chicago, Chicago, IL 60657, USA; 4 Department of Radiotherapy and Oncology, Victoria Hospital, Candos, Quatre Bornes 72259, Mauritius; 5 The University of Arizona College of Medicine at South Campus, Tucson, AZ 85713, USA

**Keywords:** lung cancer, thoracic surgery, non-small cell lung cancer, prognosis, marital status

## Abstract

Objectives: To study how marital status influences overall survival (OS) in patients with stage IA non-small cell lung cancer (NSCLC). And whether the result is valid in different time periods.

Materials and Methods: We retrospectively analyzed 55,207 cases of stage IA NSCLC from 1995 to 2015 in the Surveillance, Epidemiology, and End Results (SEER) database. Marital status was classified as follows: married or with unmarried/domestic partner (MR/W.P), divorced or separated (DV/SP), widowed (WD), and single (never married). Patients diagnosed in 1995-2005 and 2006-2015 were analyzed separately as groups 1 and 2, respectively, to validate the results. Within each group, age-stratified demographic, clinicopathologic features, and OS were compared among different marital statuses.

Results and Conclusions: A total of 55,207 cases were included (group 1 n=20,223, group 2 n=34,984). From 1995-2005 to 2006-2015, median OS was prolonged significantly in all patients besides the DV/SP subgroup. In general, being MR/W.P was associated with the lowest relative risk of death in the study population (Group 1, HR= 0.854, 95%CI: 0.816-0.893; Group 2, HR = 0.799, 95%CI: 0.758-0.842). Meanwhile, OS of DV/SP and widowed patients was similar. In group 2, being single was associated with lower risk of death beyond 60-year-old.

## INTRODUCTION

Socioeconomic factors have been actively investigated for their impact on cancer prognosis and potential to facilitate novel public health interventions. Among these factors, marital status has been found to be related to survival outcome in breast cancer [1], renal cell carcinoma [2], bladder urothelial carcinoma [3], esophageal cancer [4], rectal cancer [5] and prostate cancer [6]. However, the effect of marital status on survival of NSCLC patients remains equivocal: some researchers found no association between marital status and survival in NSCLC [7, 8], while others concluded that marital status affected overall survival (OS) of stage IV lung cancer [9] and being married was associated with lower risk of death [10].

Possible explanations of inconsistent findings in previous studies include varied sample size, age, social background, tumor malignancy and treatment models. Compared to patients with advanced cancer, Stage IA NSCLC patients usually have longer survival times and are less likely to undergo therapies other than surgical resection, e.g., chemotherapy and radiation, with debilitating side effects. Therefore, non-medical factors, such as marriage, may play more important roles in their prognosis.

Thus far, the effect of marital status on OS of stage IA NSCLC remains unclear. Meanwhile, there is neither literature comparing the effect of marital status on OS at different time periods nor in different age strata. Therefore, our primary aim was to study the impact of marital status on OS of stage IA NSCLCs patients of different ages and during different time periods in the SEER database.

## RESULTS

### Patient baseline characteristics

A total of 55,207 eligible stage IA NSCLC patients were included in this study (group 1, n = 20223 and group 2, n=34,984). Among them, 12270(60.7%)/ 20,593 (58.9%) were married, 2242 (11.1%)/ 4,366 (12.5%) were divorced, 144(0.7%)/314 (0.9%) were separated, 3780(18.7%)/ 5,748 (16.4%) were widowed, and 1,787 (8.8%)/3,897 (11.1%) were single in group 1 and group 2 respectively. Their baseline characteristics and the relationships between marital status and each variable are summarized in Tables 1, 2.

**Table 1:** Patient characteristics.

	**SEER database (1995-2005)** **group 1 N (%)**	**SEER database (2006-2015)** **group 2 N (%)**
**N**	20223	34984
**Age, (Mean ± SD), years**	67.44±10.01	68.07±9.87
**Sex**		
Female	10986 (54.3)	19930 (57.0)
Male	9237 (45.7)	15054 (43.0)
**Race**		
Black	1495 (7.4)	2671 (7.6)
Other*	1071 (5.3)	2186 (6.3)
White	17657 (87.3)	30127 (86.1)
**Grade**		
I (Well differentiated)	3078 (15.2)	8381 (24.0)
II (Moderately differentiated)	7614 (37.7)	14693 (42.0)
III (Poorly differentiated)	5960 (29.5)	8111 (23.2)
IV (Undifferentiated)	610 (3.0)	430 (1.2)
Unknown	2961 (14.6)	3369 (9.6)
**Marital status**		
Married	12270 (60.7)	20593 (58.9)
Unmarried	0 (0.0)	66 (0.2)
Divorced	2242 (11.1)	4366 (12.5)
Separated	144 (0.7)	314 (0.9)
Widowed	3780 (18.7)	5748 (16.4)
Single	1787 (8.8)	3897 (11.1)
**Tumor Size (Mean±SD), mm**	19.26±6.72	17.84±6.58

N, number of patients; SD, Standard deviation; *, includes American Indian/Alaska Native, Asian/Pacific Islander, and unknown; Unmarried, had unmarried or domestic partner.

**Table 2:** Demographic characteristics of lung cancer by marital status.

	**Married n(%)**	**Unmarried n(%)**	**Divorced n(%)**	**Separated n(%)**	**Widowed n(%)**	**Single n(%)**	**Total**
**Group 1**	**12270(60.7)**	**0(0.0)**	**2242(11.1)**	**144(0.7)**	**3780(18.7)**	**1787(8.8)**	**20223(100)**
**Race**							
Black	641(42.9)		**233(15.6)**	51(3.4)	262(17.5)	**308(20.6)**	1495(100)
Other*	734(68.5)		67(6.3)	6(0.6)	174(16.2)	90(8.4)	1071(100)
White	**10895(61.7)**		1942(11.0)	87(0.5)	3344(18.9)	1389(7.9)	17657(100)
**Sex**							
Female	5433(49.5)		**1474(13.4)**	76(0.7)	**3032(27.6)**	971(8.8)	10986(100)
Male	**6837(74.0)**		768(8.3)	68(0.7)	748(8.1)	816(8.8)	9237(100)
**Grade**							
I	1940(63.0)		309(10.0)	23(0.7)	556(18.1)	250(8.1)	3078(100)
II	4541(59.6)		920(12.1)	49(0.6)	1433(18.8)	671(8.8)	7614(100)
III	3608(60.5)		632(10.6)	47(0.8)	1154(19.4)	519(8.7)	5960(100)
IV	382(62.6)		69(11.3)	3(0.5)	108(17.7)	48(7.9)	610(100)
Unknown	1799(60.8)		312(10.5)	22(0.7)	529(17.9)	299(10.1)	2961(100)
**Age (mean± SD, years)**	**66.96(±9.67)**		64.37(±9.21)	63.04(±9.78)	**73.37(±7.51)**	62.40(±11.97)	67.44(±10.01)
**Age (median, years)**	**68**		65	64	**74**	63	69
**tumor size (n)**	12269		2241	144	3780	1785	20219
tumor size (mean± SD, mm)	19.27(± 6.70)		19.00(±6.73)	19.13(± 6.73)	19.38(± 6.71)	19.26(± 6.83)	**19.26(± 6.72)**
tumor size (median, mm)	20		20	20	20	20	20
**Group 2**	**20593(58.9)**	**66(0.2)**	**4366(12.5)**	**314(0.9)**	**5748(16.4)**	**3897(11.1)**	**34984(100)**
**Race**							
Black	1076(40.3)	4(0.1)	456(17.1)	74(2.8)	393(14.7)	668(25)	2671(100)
Other*	**1521(69.6)**	4(0.2)	155(7.1)	23(1.1)	292(13.4)	191(8.7)	2186(100)
White	17996(59.7)	58(0.2)	3755(12.5)	217(0.7)	5063(16.8)	3038(10.1)	30127(100)
**Sex**							
Female	9913(49.7)	39(0.2)	**2859(14.3)**	178(0.9)	**4618(23.2)**	2323(11.7)	19930(100)
Male	**10680(70.9)**	27(0.2)	1507(10.0)	136(0.9)	1130(7.5)	1574(10.5)	15054(100)
**Grade**							
I	5110(61.0)	17(0.2)	965(11.5)	63(0.8)	1303(15.5)	923(11.0)	8381(100)
II	8590(58.5)	27(0.2)	1868(12.7)	135(0.9)	2448(16.7)	1625(11.1)	14693(100)
III	4636(57.2)	14(0.2)	1079(13.3)	77(0.9)	1388(17.1)	917(11.3)	8111(100)
IV	249(57.9)	0(0.0)	70(16.3)	3(0.7)	65(15.1)	43(10.0)	430(100)
Unknown	2008(59.6)	8(0.2)	384(11.4)	36(1.1)	544(16.1)	389(11.5)	3369(100)
**Age (mean± SD, years)**	**67.78(±9.45)**	64.15(±10.14)	66.28(±8.75)	62.68(±9.65)	**74.20(±7.75)**	63.07(±11.52)	68.07(±9.87)
**Age (media, years)**	69	66	67	64	75	64	69
**tumor size (n)**	20582	66	4361	314	5745	3894	34962
tumor size (mean± SD, mm)	17.83(±6.55)	16.67(±6.63)	17.70(±6.58)	18.04(±6.67)	18.12(±6.59)	17.62(±6.67)	**17.84(±6.58)**
tumor size (media, mm)	18	16	17	18	18	17	18

*, includes American Indian/Alaska Native, Asian/Pacific Islander, and unknown. Unmarried, had unmarried or domestic partner.

Grade I, Well differentiated; II, Moderately differentiated; III, Poorly differentiated; IV, Undifferentiated.

Patients in the married group had a higher proportion of men and more often were White or American Indian/Alaska Native or Asian/Pacific Islander. Widowed people were more likely to be women, with a higher median age (74 years old). 66 patients from group 2 and no patients from group 1 have had unmarried or domestic partners. Married or has unmarried or domestic partners were considered the same group (MR/W.P), because these patients all had partners in life. Interestingly, the mean tumor size was smaller in group 2 than in group 1 (17.84 ± 6.58mm vs 19.26 ±6.72 mm).

### Impact of marital status on OS

Kaplan-Meier analysis and log-rank test revealed that impact of marital status on OS differs between the age-strata and may differ in different time period.

**Figure 1:**
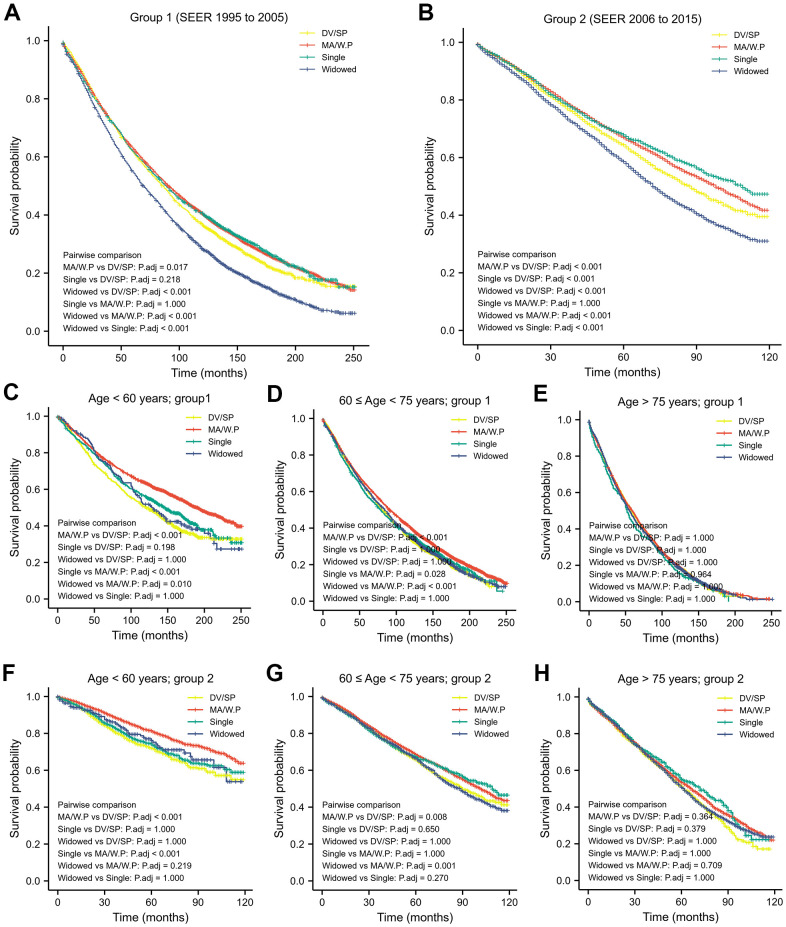
Kaplan-Meier analysis and log-rank test revealed that impact of marital status on OS varies in different time period (**A**, **B**) and in different age-strata (**C**–**H**).

Regardless of age, the widows had the worst OS, while patients being MR/W.P had the highest OS (in group1, 2, *p* < 0.05; Figure 1A, 1B). Besides, OS of the widows was similar to the DV/SP after stratification (in group 1, 2; Figure 1C–1H).

In patients younger than 60-years old, OS in the MA/W.P was statistically longer than other 3 marital statuses (group 1, 2; Table 3 and Figure 1C, 1F).

**Table 3:** Survival over marital status in different age-group within different time period.

		***N***	**Median OS** **(Months)**	**Median age** **(Years)**	**Log-rank test for equality of survival function (*p*)**			
					**DV/SP**	**MA/W.P**	**Single**	**Widowed**
age < 60 years	**group1**							
	DV/SP	695	121.0	54(50,57)		0.000	0.021	0.456
	MA/W.P	2598	**191.0**	54(50,57)	0.000		0.000	0.002
	Single	653	147.0	52(47,56)	0.021	0.000		0.443
	Widowed	176	128.0	56(54,58)	0.456	0.002	0.443	
	**group2**							
	DV/SP	1084	NR	55(52,58)		0.000	0.200	0.296
	MA/W.P	3754	NR	55(50,57)	0.000		0.000	0.036
	Single	1333	NR	54(49,57)	0.200	0.000		0.732
	Widowed	233	NR	57(53,58)	0.296	0.036	0.732	
60 ≤ age < 75 years	**group1**							
	DV/SP	1367	81.0	66(63,70)		0.000	0.738	0.685
	MA/W.P	6832	91.0	68(64,71)	0.000		0.005	0.000
	Single	868	79.0	67(63,70)	0.738	0.005		0.977
	Widowed	1819	84.0	70(66,72)	0.685	0.000	0.977	0.685
	**group2**							
	DV/SP	2746	90.0	67(64,70)		0.001	0.108	0.721
	MA/W.P	11729	102.0	68(64,71)	0.001		0.471	0.000
	Single	2009	109.0	67(63,70)	0.108	0.471		0.045
	Widowed	2502	89.0	69(66,72)	0.721	0.000	0.045	
age ≥ 75 years	**group1**							
	DV/SP	324	57.0	78(76,80)		0.828	0.377	0.906
	MA/W.P	2840	59.0	78(76,80)	0.828		0.161	0.482
	Single	266	52.0	78(76,81)	0.377	0.161		0.272
	Widowed	1785	55.0	79(77,82)	0.906	0.482	0.272	
	**group2**							
	DV/SP	850	62.0	78(76,80)		0.061	0.063	0.384
	MA/W.P	5176	66.0	78(76,81)	0.061		0.513	0.118
	Single	555	72.0	79(76,82)	0.063	0.513		0.167
	Widowed	3013	62.0	79(77,83)	0.384	0.118	0.167	

MR/W.P, married or had unmarried or domestic partner; DV/SP, divorced/separated.

From 60- to 75-year-old, OS of the MA/W.P was better than those with marriage loss (DV/SP, widows) in 1995-2005 and in 2006-2015. OS of the singles was shorter than MA/W.P in 2006-2015 (group 1), however, was improved in 2006-2015(group 2) and became similar to OS of the MA/W.P.

Starting at age 75 years, the OS was not distinct among older patients from different marital statuses (Table 3 and Figure 1E, 1H).

### Relative risk of death over marital status in different age strata

Univariate and multivariate Cox proportional hazards regression analysis demonstrated that marital status, gender, race, differentiation of tumor, and tumor size were independent prognostic factor for OS. MR/W.P patients had lower relative risk than widowed patients (Table 4) in group 1 (HR: 0.854, 95%CI: 0.816-0.893, *p* < 0.001), which also held true in group 2 (HR: 0.799, 95%CI: 0.758-0.842, *p* < 0.001) after adjustment for gender, age, race, differentiation, and tumor size.

**Table 4:** Univariate and multivariate survival analysis for evaluating the influence of marital status on OS in SEER database.

**Characteristics**	**Univariate analysis**				**Multivariate analysis**			
	**Group1** **HR (95% CI)**	**P**	**Group2** **HR (95% CI)**	**P**	**Group1** **HR (95% CI)**	**P**	**Group2** **HR (95% CI)**	**P**
**Marital status**		<0.001		<0.001		<0.001		<0.001
MA/W.P	0.729 (0.700-0.760)	<0.001	0.733 (0.699-0.770)	<0.001	0.854 (0.816-0.893)	<0.001	0.799 (0.758-0.842)	<0.001
DV/SP	0.789 (0.744-0.836)	<0.001	0.821 (0.769-0.877)	<0.001	1.087 (1.023-1.155)	0.007	1.010 (0.944-1.081)	0.770
Single	0.725 (0.679-0.775)	<0.001	0.699 (0.650-0.752)	<0.001	1.041 (0.972-1.115)	0.247	0.924 (0.855-0.998)	0.043
Widowed	Reference		Reference		Reference		Reference	
**Sex**		<0.001		<0.001		<0.001		<0.001
Female	0.723 (0.700-0.746)	<0.001	0.638 (0.615-0.662)	<0.001	0.726 (0.701-0.751)	<0.001	0.642 (0.617-0.668)	<0.001
Male	Reference		Reference		Reference		Reference	
**Race**		<0.001		<0.001		<0.001		<0.001
Black	1.111 (1.046-1.180)	0.001	0.967 (0.900-1.039)	0.364	1.178 (1.108-1.252)	<0.001	1.049 (0.975-1.128)	0.204
Others*	0.765 (0.708-0.826)	<0.001	0.640 (0.582-0.704)	<0.001	0.756 (0.700-0.816)	<0.001	0.704 (0.640-0.774)	<0.001
White	Reference		Reference		Reference		Reference	
**Grade**		<0.001		<0.001		<0.001		<0.001
I	0.996 (0.935-1.061)	0.897	0.599 (0.554-0.648)	<0.001	0.912(0.855-0.972)	0.004	0.604 (0.558-0.653)	<0.001
II	1.435 (1.362-1.512)	<0.001	1.029 (0.962-1.100)	0.408	1.272(1.207-1.341)	<0.001	0.962 (0.899-1.029)	0.256
III	1.726 (1.636-1.820)	<0.001	1.326 (1.236-1.421)	<0.001	1.500(1.422-1.583)	<0.001	1.213 (1.131-1.301)	<0.001
IV	1.699 (1.539-1.875)	<0.001	1.335 (1.146-1.557)	<0.001	1.546(1.400-1.707)	<0.001	1.286 (1.103-1.499)	0.001
Unknown	Reference		Reference		Reference		Reference	
**Tumor Size (mm)**	1.015 (1.012-1.017)	<0.001	1.013(1.010-1.016)	<0.001	1.005 (1.002-1.007)	<0.001	1.005 (1.002-1.008)	<0.001
**Age**	1.047(1.045-1.049)	<0.001	1.041 (1.038-1.043)	<0.001	1.047 (1.045-1.049)	<0.001	1.039 (1.036-1.041)	<0.001

*, includes American Indian/Alaska Native, Asian/Pacific Islander, and unknown; Grade I, Well differentiated; II, Moderately differentiated; III, Poorly differentiated; IV, Undifferentiated. MR/W.P, married or had unmarried or domestic partner; DV/SP, divorced/separated.

Impact of marital status on OS differs within in different age-strata and the median of age differs in each marital status, especially in widowed, whose median age was much higher than other patients. Therefore, to estimate adjust relative risk over marital status, we performed subgroup analysis within each age strata. All known prognostic factors including age were included in the multivariate cox regression analysis (Table 4). Widowed patients was the reference group, MA/W.P was associated with significant lower relative risk regardless of time and age, even in elderlies > 75-year old. Single, interestingly, was associated with lower risk similar to MA/W.P for patients ≥ 60 years-old and diagnosed as stage 1A NSCLC after 2005 (Table 5, group 2).

**Table 5:** Multivariate survival analysis for dying associated with marital status stratified by age within different time period in SEER database.

	**Age < 60 years**				**60 ≤ age < 75 years**				**Age ≥ 75 years**			
	**Group1**		**Group2**		**Group1**		**Group2**		**Group1**		**Group2**	
	**HR (95% CI)**	**P**	**HR (95% CI)**	**P**	**HR (95% CI)**	**P**	**HR (95% CI)**	**P**	**HR (95% CI)**	**P**	**HR (95% CI)**	**P**
**Marital status**		**0.000**		**0.000**		**0.000**		**0.000**		**0.013**		**0.000**
MA/W.P	0.783 (0.639, 0.960)	**0.018**	0.717 (0.537, 0.956)	**0.024**	0.831 (0.780, 0.885)	**0.000**	0.794 (0.734, 0.860)	**0.000**	0.904 (0.843, 0.969)	**0.004**	0.835 (0.775, 0.900)	**0.000**
DV/SP	1.152 (0.929, 1.429)	0.198	1.089 (0.805, 1.472)	0.581	1.055 (0.973, 1.144)	0.198	0.970 (0.880, 1.069)	0.543	0.995 (0.877, 1.128)	0.935	1.032 (0.921, 1.157)	0.588
Single	1.043 (0.836, 1.302)	0.709	1.061 (0.785, 1.434)	0.701	0.995 (0.905, 1.094)	0.922	0.873 (0.782, 0.975)	**0.016**	1.040 (0.908, 1.191)	0.572	0.850 (0.735, 0.982)	**0.027**
Widowed	Reference		Reference		Reference		Reference		Reference		Reference	

Adjusted by Age, tumor size, grade, race and sex within each age-group.

Others*, includes American Indian/Alaska Native, Asian/Pacific Islander, and unknown.

### Impact of marital status on OS during the last two decades

From 1995 to 2005, the median OS of stage IA NSCLC patients was 86 months, while from 2006 to 2015, the median OS was 92months (Table 6, *p* < 0.001). OS was prolonged significantly in both widowed (*p* = 0.018), MR/W.P and single patients during the past 20 years *(p* < 0.001). However, such improvements in median OS were not found in DV/SP patients (median OS was 86 and 88 months respectively, *p* = 0.171).

**Table 6:** Median OS and marital status in different time periods.

	**Median OS(months)**		**95% CI**		***p***
			**Low**	**Up**	
DV/SP	group1	86.000	81.433	90.567	
	group2	88.000	83.263	92.737	0.171
MA/W.P	group1	91.000	88.661	93.339	
	group2	98.000	95.321	100.679	0.000
Single	group1	89.000	82.808	95.192	
	group2	109.000	NA	NA	0.000
Widowed	group1	70.000	66.720	73.280	
	group2	72.000	69.332	74.668	0.018
total	group1	86.000	84.308	87.692	
	group2	92.000	90.095	93.905	0.000

MR/W.P, married or had unmarried or domestic partner; DV/SP, divorced/separated.

## DISCUSSION

Several studies have found that cancer patients in marriage are more likely to live longer [11]. Other researchers have reported greater benefits of marriage on conditional relative survival in cancer at early stages [12]. However, the association between marital status and OS of NSCLC patients remains controversial [8, 13–15]. Such controversy may partially arise from treatment complexity in advanced-stage cancer. In order to minimize confounding issues such as differing chemo- and radiotherapy regimens, we focused on stage IA NSCLCs, for which surgical resection alone is the only recommended treatment. As a result, the impact of marital status on OS can be explored while minimizing confounding medical factors.

In the last two decades, cancer screening methods, surgical strategies, population characteristics, social paradigms, and economic factors have evolved significantly in the United States. Hence, analysis was performed separately in two consecutive time periods to validate the result. Another important factor is age, which can be both a confounder and an effect modifier. Theoretically, people are more capable of recovering from marriage failure when they are young. Psychosocial interventions may have an important effect on survival and these interventions appear to be more effective in patients who are unmarried, older, attend Cognitive-Behavioral Therapy and have early-stage cancer 21. Since the World Health Organization categorizes age beyond 60-year-old as elderly, median age of the widows was 74-year old, patients beyond 74 had similar median age in different marital status, and using 60, 65 or 70 as boundary of stratified analysis would yield similar result (impact of marital status on OS), we decided to separate patients into three subgroups (age ≤60, 60 ≤ age < 75, age ≥ 75 years).

Although some of our findings corroborated previous publications, the following new findings were identified in our study.

First, we revealed a significantly prolonged OS during the last two decades except for the DV/SP patients: Median OS was prolonged significantly in single patients (from 89 to 109 months, *p* <0.001) and in widowed patients (from 70 months to 72months, *p* = 0.018). This can be explained mainly by progress in medical science. However, median OS of DV/SP patients did not show a statistically significant increase over the 20 years. Many factors, including lifestyle and weak social support systems, may contribute to this phenomenon. These socioeconomic factors [16] may impact cancer prognosis and even outweigh the survival benefits from medical progress. As the global population ages, further studies are needed to explain and address this problem.

Nevertheless, this is the first-time failed marriage (DV/SP) was shown to potentially offset the survival benefits from medical progress in the last decade in stage IA NSCLC patients, which can contribute to current literatures.

Second, this study not only verified the conclusion that being married is associated with lower relative risk of death in cancer patients [4, 7, 15, 17–19] but also validated protective effect of MA/W.P in cohort from different time periods and in different age group. We reported, for the first-time, possible protective role of being single (Table 4). which seems only present in patients ≥ 60 years and received diagnosis after 2005. As time goes by, populations of different marital status may have different characteristics. And how marital status affects cancer survival cloud also change. Therefore, further studies are required to understand the mechanisms of the evolving phenomenon and to provide updated reference to personalized care.

Finally, this is the first study revealing that the impact of marital status on patient survival varies significantly by age-group. Previous studies on the impact of widowhood on survival have had conflicting results. One study of Floridian patients with lung cancer revealed longer survival in married and widowed patients than in DV/SP and single patients [20]. Another Japanese study of 1,230 NSCLC patients demonstrated that widowed patients had the worst survival among married, separated, divorced and single male patients [14]. One study of 5,898 NSCLC patients from Mayo Clinic Lung Cancer Cohort found no statistically significant difference in survival among widowed, married, single and divorced patients, while subgroup analysis found stage IA widowed patients had a shorter survival [8]. Age difference may contribute to such controversy, for instance median age of the widows is 6-12 years elder than other marital status. In our study, after adjusting by age and other factors, being widowed and the DV/SP patients had similar OS shorter than the MA/W.P. One possible explanation is their social networks and may rely more on their families for support. The passing away of a loved one or marriage loss may lead to psychological trauma, less emotional and financial support.

Survival disparities based on marital status could also be attributed to the influence of family on the patient’s treatment decisions. Previous studies demonstrated that the family was involved in the decision-making process for most patients first-time diagnosed with lung cancer [21]. Unmarried patients, when diagnosed with staged IA NSCLC, more commonly refused surgery and came from lower socioeconomic backgrounds [22]. In addition, widowed and divorced patients preferred less aggressive cancer therapy [8] and were less likely to receive first-line surgical treatment [23].

This retrospective study has limitations: first, the potential for selection bias exists, as the sample population is mainly U.S based. Although robust data from other countries was not accessible, validation was performed using another group of patients with a different year of diagnosis. Moreover, the large sample size supports these findings as extrapolatable to the general population. Second, although both regression and stratification were used to control for confounding variables, unknown confounders may still exist, including comorbidities and other social variables, especially retirement. Patients were stratified by age older or younger than 60 years, which is a common age of retirement. However, retirement could not directly be controlled for, as this information was absent in the records.

In summary, this is the first age-stratified study about marital status and survival on early-stage NSCLC using historical cohort for validation. Being married or having an unmarried partner was associated with a significant lower relative risk. In contrast, marriage loss (widowed, DV/SP) were at a significantly higher risk of death. The progress of medical science in the preceding decades did not benefit survival of patients with marriage loss. Future studies could be designed with input from multidisciplinary specialists, e.g., psychologists and geriatric physicians, to further elucidate the underlying mechanisms and establish tangible interventions to address these survival disparities among individuals with different marital statuses.

## MATERIALS AND METHODS

### SEER database

Permission to access SEER research-data files was obtained using reference number 16139-Nov2017. As the SEER database does not require informed patient consent, this study was exempted from the ethical approval requirements of the Institutional Review Board (IRB). NSCLC cases between 1995 and 2015 in the SEER public access database and their corresponding details were retrieved with the use of SEER*Stat version 8.3.5 software. Cases from 1995 to 2005 were designated as the test data set, while cases from 2006-2015 were designated as the validation data set.

### Patient selection

A total of 55,207 cases were obtained using the following inclusion criteria: (1) Underwent surgery for first-time diagnosed primary NSCLC. (2) Clinically and pathologically staged T1N0M0 (3) Tumor size ≤ 3cm (or recorded as T1 on diagnosis). (4) With known marital status. Patients without marital status or survival data were excluded. Eligible patients were categorized by marital status, age at diagnosis, race, sex, and grade and size of tumor (millimeter, mm). Marital status at diagnosis was the primary variable of interest and classified as married or with unmarried/domestic partner (MR/W.P), divorced or separated (DV/SP), widowed(WD), and single (never married).

### Statistical analyses

Patients were assigned either to group 1 (1995-2005, test group) or group 2 (2006-2015, validate group) based on year of diagnosis. Non-normally distributed continuous covariables were categorized and analyzed using the Non-parametric Mann-Whitney U test. Normally distributed continuous covariables were analyzed using the Student’s t-test and presented as median ± standard deviation (SD). Data with categorical covariates were analyzed using the Pearson's chi-squared test. OS was estimated with the Kaplan-Meier method; differences were calculated using the log rank test and Benjamini multiple hypothesis testing. Univariate and multivariate Cox proportional hazards regression models were built to analyze hazard ratios of different prognostic variables. These analyses were performed with SPSS soft-ware version 23.0 (IBM Corporation, Armonk, NY, USA), and R statistical version 3.6.3 using ‘survminer’ and ‘survival’ packages. The *p*-value<0.05 (2-sided) was considered statistically significant.

## References

[1] Liu L, Chi YY, Wang AA, Luo Y. Marital Status and Survival of Patients with Hormone Receptor-Positive Male Breast Cancer: A Surveillance, Epidemiology, and End Results (SEER) Population-Based Study. Med Sci Monit. 2018; 24:3425–41. 10.12659/MSM.910811 29795054 PMC5994964

[2] Li Y, Zhu MX, Qi SH. Marital status and survival in patients with renal cell carcinoma. Medicine (Baltimore). 2018; 97:e0385. 10.1097/MD.0000000000010385 29668592 PMC5916654

[3] Niu Q, Lu Y, Wu Y, Xu S, Shi Q, Huang T, Zhou G, Gu X, Yu J. The effect of marital status on the survival of patients with bladder urothelial carcinoma: A SEER database analysis. Medicine (Baltimore). 2018; 97:e11378. 10.1097/MD.0000000000011378 30024509 PMC6086512

[4] Du L, Kim JJ, Chen B, Zhu S, Dai N. Marital status is associated with superior survival in patients with esophageal cancer: a Surveillance, Epidemiology, and End Results study. Oncotarget. 2017; 8:95965–72. 10.18632/oncotarget.21609 29221179 PMC5707073

[5] Li Z, Wang K, Zhang X, Wen J. Marital status and survival in patients with rectal cancer: A population-based STROBE cohort study. Medicine (Baltimore). 2018; 97:e0637. 10.1097/MD.0000000000010637 29718875 PMC6392664

[6] Huang TB, Zhou GC, Dong CP, Wang LP, Luan Y, Ye JT, Gu X, Yao XD, Zheng JH, Ding XF. Marital status independently predicts prostate cancer survival in men who underwent radical prostatectomy: An analysis of 95,846 individuals. Oncol Lett. 2018; 15:4737–44. 10.3892/ol.2018.7964 29552113 PMC5840566

[7] Saito-Nakaya K, Nakaya N, Fujimori M, Akizuki N, Yoshikawa E, Kobayakawa M, Nagai K, Nishiwaki Y, Tsubono Y, Uchitomi Y. Marital status, social support and survival after curative resection in non-small-cell lung cancer. Cancer Sci. 2006; 97:206–13. 10.1111/j.1349-7006.2006.00159.x 16542217 PMC11159180

[8] Jatoi A, Novotny P, Cassivi S, Clark MM, Midthun D, Patten CA, Sloan J, Yang P. Does marital status impact survival and quality of life in patients with non-small cell lung cancer? Observations from the mayo clinic lung cancer cohort. Oncologist. 2007; 12:1456–63. 10.1634/theoncologist.12-12-1456 18165623

[9] Varlotto JM, Voland R, McKie K, Flickinger JC, DeCamp MM, Maddox D, Rava P, Fitzgerald TJ, Graeber G, Rassaei N, Oliveira P, Ali S, Belani C, et al. Population-based differences in the outcome and presentation of lung cancer patients based upon racial, histologic, and economic factors in all lung patients and those with metastatic disease. Cancer Med. 2018; 7:1211–20. 10.1002/cam4.1430 29533006 PMC5911616

[10] Aizer AA, Chen MH, McCarthy EP, Mendu ML, Koo S, Wilhite TJ, Graham PL, Choueiri TK, Hoffman KE, Martin NE, Hu JC, Nguyen PL. Marital status and survival in patients with cancer. J Clin Oncol. 2013; 31:3869–76. 10.1200/JCO.2013.49.6489 24062405 PMC4878087

[11] Wu W, Fang D, Shi D, Bian X, Li L. Effects of marital status on survival of hepatocellular carcinoma by race/ethnicity and gender. Cancer Manag Res. 2018; 10:23–32. 10.2147/CMAR.S142019 29379317 PMC5757210

[12] Merrill RM, Johnson E. Benefits of marriage on relative and conditional relative cancer survival differ between males and females in the USA. J Cancer Surviv. 2017; 11:578–89. 10.1007/s11764-017-0627-y 28770444

[13] Siddiqui F, Bae K, Langer CJ, Coyne JC, Gamerman V, Komaki R, Choy H, Curran WJ, Watkins-Bruner D, Movsas B. The influence of gender, race, and marital status on survival in lung cancer patients: analysis of Radiation Therapy Oncology Group trials. J Thorac Oncol. 2010; 5:631–9. 10.1097/jto.0b013e3181d5e46a 20432520

[14] Saito-Nakaya K, Nakaya N, Akechi T, Inagaki M, Asai M, Goto K, Nagai K, Nishiwaki Y, Tsugane S, Fukudo S, Uchitomi Y. Marital status and non-small cell lung cancer survival: the Lung Cancer Database Project in Japan. Psychooncology. 2008; 17:869–76. 10.1002/pon.1296 18033697

[15] Yang H, Li X, Shi J, Fu H, Yang H, Liang Z, Xiong H, Wang H. A nomogram to predict prognosis in patients undergoing sublobar resection for stage IA non-small-cell lung cancer. Cancer Manag Res. 2018; 10:6611–26. 10.2147/CMAR.S182458 30584357 PMC6284539

[16] Mirosevic S, Jo B, Kraemer HC, Ershadi M, Neri E, Spiegel D. “Not just another meta-analysis”: Sources of heterogeneity in psychosocial treatment effect on cancer survival. Cancer Med. 2019; 8:363–73. 10.1002/cam4.1895 30600642 PMC6346264

[17] Qiu Y, Jiang J, Zhang M, Qin Y. Positive PD-L1 expression is predictive for patients with advanced EGFR wild-type non-small cell lung cancer treated with gemcitabine and cisplatin. Oncol Lett. 2019; 18:161–8. 10.3892/ol.2019.10302 31289485 PMC6539442

[18] Osazuwa-Peters N, Christopher KM, Cass LM, Massa ST, Hussaini AS, Behera A, Walker RJ, Varvares MA. What’s Love Got to do with it? Marital status and survival of head and neck cancer. Eur J Cancer Care (Engl). 2019; 28:e13022. 10.1111/ecc.13022 30784126

[19] Alvi MA, Wahood W, Huang AE, Kerezoudis P, Lachance DH, Bydon M. Beyond Science: Effect of Marital Status and Socioeconomic Index on Outcomes of Spinal Cord Tumors: Analysis From a National Cancer Registry. World Neurosurg. 2019; 121:e333–43. 10.1016/j.wneu.2018.09.103 30261382

[20] Tannenbaum SL, Zhao W, Koru-Sengul T, Miao F, Lee D, Byrne MM. Marital status and its effect on lung cancer survival. Springerplus. 2013; 2:504. 10.1186/2193-1801-2-504 25674396 PMC4320128

[21] Hobbs GS, Landrum MB, Arora NK, Ganz PA, van Ryn M, Weeks JC, Mack JW, Keating NL. The role of families in decisions regarding cancer treatments. Cancer. 2015; 121:1079–87. 10.1002/cncr.29064 25708952 PMC4368490

[22] Ou SH, Zell JA, Ziogas A, Anton-Culver H. Low socioeconomic status is a poor prognostic factor for survival in stage I nonsmall cell lung cancer and is independent of surgical treatment, race, and marital status. Cancer. 2008; 112:2011–20. 10.1002/cncr.23397 18361399

[23] Dalton SO, Steding-Jessen M, Jakobsen E, Mellemgaard A, Østerlind K, Schüz J, Johansen C. Socioeconomic position and survival after lung cancer: Influence of stage, treatment and comorbidity among Danish patients with lung cancer diagnosed in 2004-2010. Acta Oncol. 2015; 54:797–804. 10.3109/0284186X.2014.1001037 25761702

